# Mortality in cancer patients with a history of cutaneous squamous cell carcinoma - a nationwide population-based cohort study

**DOI:** 10.1186/1471-2407-12-126

**Published:** 2012-03-29

**Authors:** Sigrun Alba Johannesdottir, Timothy L Lash, Annette Østergaard Jensen, Dóra Körmendiné Farkas, Anne Braae Olesen

**Affiliations:** 1 Department of Clinical Epidemiology, Aarhus University Hospital, Olof Palmes Allé 43-45, 8200 Aarhus N, Denmark; 2 Department of Dermatology, Aarhus University Hospital, P.P. Ørumsgade 11, 8000 Aarhus C, Denmark

## Abstract

**Background:**

Cutaneous squamous cell carcinoma (SCC) is associated with underlying immunosuppression, so it may be a prognostic marker in patients with subsequent cancer. We therefore conducted a nationwide population-based Danish cohort study to evaluate whether a history of cutaneuos SCC has prognostic impact in patients with one of the following index cancers: non-Hodgkin's lymphoma (NHL), or cancer of the lung, colon, rectum, breast, or prostate.

**Methods:**

We used Danish medical databases, which cover the entire Danish population of 5.6 million inhabitants and linked them using the unique personal identification number assigned to all Danish residents. From 1982 through 2003, we identified 745 index cancer patients with and 79,143 without previous cutaneous SCC. Using Cox proportional hazards regression, we calculated adjusted mortality rate ratios (MRRs) with 95% confidence intervals (CIs).

**Results:**

Overall, previous SCC was associated with an increased mortality of cancer (MRR 1.13, 95% CI: 1.04-1.23). When examining index cancers separately, increased MRRs were found for cancer of the lung (MRR 1.23, 95% CI: 1.05-1.43), colon (MRR 1.13, 95% CI: 0.92-1.40), rectum (MRR 1.29, 95% CI: 1.00-1.67), breast (MRR 1.09, 95% CI: 0.82-1.43), and NHL (MRR 1.09, 95% CI: 0.81-1.47), but not for prostate cancer (MRR 0.99, 95% CI: 0.83-1.18).

**Conclusions:**

Our results suggest that previous cutaneous SCC is associated with poor prognosis of some cancers. This finding stresses the importance of adherence to the existing recommendations of screening, diagnosis, and treatment of cancer in patients with a history of SCC.

## Background

Non-melanoma skin cancer (NMSC)--basal cell carcinoma (BCC) and squamous cell carcinoma (SCC) [1]--is associated with an increased risk of developing both subsequent NMSC [2] and other malignancies compared with the general population [3-5]. Common risk factors have been proposed as the cause, for example, ultraviolet radiation (UVR) and immune incompetence [6,7]. In particular, cutaneous SCC is known to be associated with immunosuppression [6,8,9], but whether it is a reliable marker for reduced immune competence that could explain these findings is unknown. Overall, five studies have found higher mortality rates in cancer patients with previous SCC [4,10-13], which also could be explained by reduced immune competence. None of the studies, however, included information on important factors such as cancer treatments and comorbidity. Therefore, taking these factors into account, while focusing on the most common types of cancers, we conducted a nationwide population-based Danish cohort study to examine whether a history of cutaneous SCC has prognostic impact in patients with a subsequent diagnosis of one of the following index cancers: non-Hodgkin's lymphoma (NHL), or cancer of the lung, colon, rectum, breast, or prostate.

## Methods

The current study was conducted using Danish medical databases, which cover the entire Danish population of 5.6 million inhabitants [14]. Accurate and unambiguous linkage of all registries was possible using the unique personal identification number assigned to all Danish residents [14].

### Study cohort

From the Danish Cancer Registry (DCR), we identified all patients aged 20 to 99 years with a first diagnosis of an index cancer from 1982 through 2003 occurring in the month after a cutaneous SCC diagnosis or later. Because of the rarity of a history of SCC of the skin among cancer patients, we chose to focus on the most common types of cancer (cancer of the lung, colon, rectum, breast, or prostate). In addition, we included NHL because of its link to immune function and for comparison with previous studies on the topic [4,10-13]. The DCR contains records of all incident malignant neoplasms in Denmark since 1943 and provides details on morphology, histology, stage of cancer at the time of diagnosis, and initial cancer therapies within four months of diagnosis [15]. All diagnostic codes used in this study are provided in the Additional file 1: Table S1.

Given abundant data in the DCR, in combination with the fact that our focus was mortality following the most common cancers in Denmark, we decided that for each selected cancer patient with a history of cutaneous SCC, we would randomly choose 100 patients with the same index cancer but without preceding SCC of the skin.

Initially, we aimed to include human immunodeficiency virus (HIV) diagnosis and previous solid organ transplantation as a measure of immune function, but due to a small numbers of patients in these categories, we excluded all patients with HIV (n = 4) or previous solid organ transplantation (n = 45). Due to a small numbers of patients with a history of cutaneous SCC in age groups 20-29 years (n = 0), 30-39 years (n = 1) and 40-49 years (n = 3), we chose not to include these age groups in the analysis.

### Mortality data

We identified all-cause death using the Danish Civil Registration System (established on April 2, 1968), which is updated daily and records all changes in vital status, date of death, and migration [14]. We identified cancer-specific death using the Danish Registry of Causes of Death, which contains information on all deaths in Denmark since 1943 [16]. We used the cause of death reported on the death certificate grouped into 14 categories as defined by the National Board of Health [17]. Each death certificate includes one underlying, and up to three immediate causes of death [16]. If any of these causes were a malignancy, we used that as the cause of death.

### Comorbidity

We obtained information on comorbid diseases from The Danish National Patient Registry, which provides information about all inpatient admissions to somatic hospitals since 1977, and all outpatient and emergency admissions since 1995 [18]. We categorized the level of comorbidity by using the Charlson Comorbidity Index (CCI) [19], an extensively studied and validated instrument used to predict risk of death from comorbid diseases [19,20]. We computed the CCI score for each study subject based on the complete hospital discharge history for at least 5 years before index cancer diagnosis, and grouped it into three levels: Low = 0, medium = 1-2, and high > 3.

According to our hypothesis cutaneous SCC is associated with poor prognosis in cancer patients due to underlying immune incompetence. We would therefore expect diseases involving the immune system or immunosuppressive therapies to be more frequent in patients with a history of SCC. To examine this, we also included a list of autoimmune diseases, as a proxy measure of immune function.

### Statistical analysis

To include information on initial cancer therapies, follow-up started four months after index cancer diagnosis, and continued until death, emigration, diagnosis of cutaneous SCC in patients without that history at diagnosis of the index cancer, end of follow-up (31 December 2008), or a maximum of 10 years, whichever came first. 130 patients who had died or were censored by start of follow-up were not included in the analysis.

Initially, we computed the frequency and proportion of covariates, number of deaths and amount of accumulated person-time within index cancer cohorts, stratified by history of cutaneous SCC. Then, we calculated crude mortality rate ratios (MRRs) with 95% confidence intervals (CIs) associating previous cutaneous SCC with mortality.

We used Cox proportional hazard regression to estimate MRRs with 95% CIs for index cancer patients with a history of cutaneous SCC compared with index cancer patients without such history adjusting for age group (50-69, 70-79, 80-89, 90-99 years), a variable that calculated the midpoint of the age group divided by exact age for each individual, gender, CCI (low, medium, high), calendar period (1982-1986, 1987-1991, 1992-1996, 1997-2001, 2002-2003), history of autoimmune disease (yes/no), stage (localized, regional, distant, unknown/missing), and the following index cancer treatments: no/symptomatic treatment, chemotherapy, radiation therapy, hormone therapy, operation, and other/missing treatment. To examine the presence of effect modification, we stratified the model on age groups, gender, CCI, and history of autoimmune disease. We also stratified mortality rates on time between SCC and index cancer diagnoses. Next, we fitted a reduced model without adjustment for stage and treatment since we hypothesized that they may be on the causal pathway linking cutaneous SCC to poor prognosis. That is, decreased immune surveillance may cause faster progression of the cancers and thereby more advanced stage at diagnosis, which in turn affects the choice of treatment. In a subanalysis, we found no substantial difference between the phenotypic variant chronic lymphocytic leukemia and other NHL types and therefore report the pooled results. All analyses were performed for both all-cause and cancer-specific death within each index cancer and overall. 476 persons, who were registered as dead in the CRS, but not in the Danish Registry of Causes of Death, were censored at the date of death in analysis for death from cancer. They did not differ from the total population with regard to exposure. Finally, we assessed the assumption of proportional hazards by graphical examination of log-log plots against log-time and found it not to be violated.

After 2003, information on cancer treatments was not available. In a subanalysis, we excluded treatment from the model, which allowed us to increase the enrollment period from 1982 through 2008, and thereby, include more patients. Lung and breast cancer was left out from this analysis, since their results were affected by adjustment for cancer treatment.

If cutaneous SCC is diagnosed shortly after an index cancer it could still be a marker of poor prognosis, causing us to underestimate the effect when including such patients in the comparison group. We therefore repeated our analysis after excluding patients receiving a diagnosis of cutaneous SCC within two years after index cancer diagnosis. This exclusion did not change the estimates.

All analyses were performed using STATA^® ^software (version 11.0, STATA, College Station, TX). Because the data are obtained from existing medical registries there are no considerable ethical aspects of this study and the study was approved by the Danish Data Protection Agency (Registration number: 2007-58-0010).

## Results

### Patient characterstics

We included 745 index cancer patients with and 79,143 without a history of cutaneous SCC. Patients with previous cutaneous SCC were older at index cancer diagnosis, were more frequently men, had their index cancer diagnosis in a more recent calendar period, and had higher comorbidity (Table 1). Furthermore, SCC patients had more frequently missing stage and treatment information, more often received no or symptomatic treatment (except in breast cancer) and had more frequently a history of any autoimmune disease (see Additional file 2: Table S2).

**Table 1:** Selected characteristics of persons diagnosed with an index cancer (cancer of the lung, colon, rectum, breast, prostate, or non-Hodgkin's lymphoma (NHL)) in Denmark 1982-2003, by history of cutaneous squamous cell carcinoma (SCC)

Characteristics	Lung cancer		Colon cancer		Rectal cancer	
	+ SCC (%)	- SCC (%)	+ SCC (%)	- SCC (%)	+ SCC (%)	- SCC (%)
*Total*	175	18,662	138	14,647	77	7,089
*Gender*						
Men	140 (80)	11,673 (63)	87 (63)	6,592 (45)	60 (78)	4,086 (58)
Women	35 (20)	6,989 (37)	51 (37)	8,055 (55)	17 (22)	3,003 (42)
*Age group (years)^a^*						
50-69	49 (28)	11,471 (61)	23 (17)	5,968 (41)	15 (19)	3,383 (48)
70-79	82 (47)	5,895 (32)	48 (35)	5,479 (37)	34 (44)	2,487 (35)
80-89	41 (23)	1,241 (6.7)	59 (43)	2,958 (20)	22 (29)	1,107 (16)
90-99	3 (1.7)	55 (0.3)	8 (5.8)	242 (1.7)	6 (7.8)	112 (1.6)
*Calendar period^b^*						
1982-1986	19 (11)	3,798 (20)	12 (8.7)	3,023 (21)	11 (14)	1,565 (22)
1987-1991	25 (14)	3,866 (21)	26 (19)	3,219 (22)	15 (19)	1,557 (22)
1992-1996	47 (27)	4,101 (22)	35 (25)	3,324 (23)	22 (29)	1,578 (22)
1997-2001	66 (38)	4,832 (26)	49 (36)	3,549 (24)	17 (22)	1,683 (24)
2002-2003	18 (10)	2,065 (11)	16 (12)	1,532 (10)	12 (16)	706 (10)
*Comorbidity level^c^*						
Low	81 (46)	12,081 (65)	81 (59)	10,806 (74)	54 (70)	5,477 (77)
Moderate	69 (39)	5,306 (28)	47 (34)	3,221 (22)	18 (23)	1,365 (19)
High	25 (14)	1,275 (6.8)	10 (7.3)	620 (4.2)	5 (6.5)	247 (3.5)

	**Breast cancer**		**Prostate cancer**		**NHL^d^**	
	**+ SCC (%)**	**- SCC (%)**	**+ SCC (%)**	**- SCC (%)**	**+ SCC (%)**	**- SCC (%)**

*Total*	113	10,418	186	21,364	56	6,963
*Gender*						
Men	-	-	186 (100)	21,364 (100)	46 (82)	3,896 (56)
Women	113 (100)	10,418 (100)	-	-	10 (18)	3,067 (44)
*Age group (years)^a^*						
50-69	25 (22)	6,298 (60)	18 (9.7)	7,014 (33)	11 (20)	3,253 (47)
70-79	38 (34)	2,569 (25)	83 (45)	9,423 (44)	21 (38)	2,492 (36)
80-89	40 (35)	1,369 (13)	77 (41)	4,61 (22)	20 (36)	1,130 (16)
90-99	10 (8.9)	182 (1.8)	8 (4.3)	308 (1.4)	4 (7.1)	88 (1.3)
*Calendar period^b^*						
1982-1986	6 (5.3)	1,866 (18)	20 (11)	3,825 (18)	8 (14)	1,261 (18)
1987-1991	18 (16)	2,029 (19)	30 (16)	4,562 (21)	10 (18)	1,392 (20)
1992-1996	28 (25)	2,469 (24)	39 (21)	4,333 (20)	13 (23)	1,659 (24)
1997-2001	44 (39)	2,806 (27)	69 (37)	5,700 (27)	15 (27)	1,876 (27)
2002-2003	17 (15)	1,248 (12)	28 (15)	2,944 (14)	10 (18)	775 (11)
*Comorbidity level^c^*						
Low	77 (68)	8,406 (81)	106 (57)	14,554 (68)	28 (50)	5,183 (74)
Moderate	29 (26)	1,667 (16)	58 (31)	5,558 (26)	20 (36)	1,517 (22)
High	7 (6.2)	345 (3.3)	22 (12)	1,252 (5.9)	8 (14)	263 (3.8)

^a^Age at index cancer diagnosis

^b^Calendar period of index cancer diagnosis

^c^Three levels of comorbidity were defined based on Charlson index scores of 0 (low), 1-2 (medium), and > 2 (high)

^d^Includes the phenotypic variant chronic lymphocytic leukemia

### Mortality

We observed shorter survival time among those with a history of cutaneous SCC within all index cancer groups, with an overall median survival time of 1.93 years (lower quartile 0.58 years; upper quartile 5.09 years) in SCC patients and 2.57 years (lower quartile 0.74 years; upper quartile 6.54 years) in patients without SCC (Additional file 2: Table S2). The most frequent cause of death was malignancy. We were not able to distinguish between NMSC subtypes for cause of death, but a total of 22 persons died of NMSC (2.95% of deaths) among the exposed and 31 (0.04% of deaths) among the unexposed patients.

Overall, a history of cutaneous SCC was associated with an increased relative rate of death from cancer (MRR 1.13, 95% CI: 1.04-1.23) (Table 2). When examining index cancers separately, increased MRRs were found for cancer of the lung (MRR 1.23, 95% CI: 1.05-1.43), colon (MRR 1.13, 95% CI: 0.92-1.40), rectum (MRR 1.29, 95% CI: 1.00-1.67), breast (MRR 1.09, 95% CI: 0.82-1.43), and NHL (MRR 1.09, 95% CI: 0.81-1.47). There was no increased rate of dying of prostate cancer (MRR 0.97, 95% CI: 0.81-1.15). The impact of covariates on the mortality rate of cancer is shown in Table 3. The stratified analysis revealed no effect modification (data not shown).

**Table 2:** Mortality rate ratios for death from cancer, associated with prior cutaneous squamous cell carcinoma in persons diagnosed with cancer of the lung, colon, rectum, breast, prostate, or non-Hodgkin's lymphoma (NHL) in Denmark 1982-2003

	Unadjusted MRR (95% CI)	Adjusted^a^ MRR (95% CI)
Lung cancer	1.27 (1.09-1.48)	1.23 (1.05-1.43)
Colon cancer	1.31 (1.06-1.61)	1.13 (0.92-1.40)
Rectal cancer	1.53 (1.19-1.98)	1.29 (1.00-1.67)
Breast cancer	1.37 (1.05-1.81)	1.09 (0.82-1.43)
Prostate cancer	1.11 (0.93-1.32)	0.97 (0.81-1.15)
NHL^b^	1.46 (1.09-1.96)	1.09 (0.81-1.47)
Overall	1.24 (1.14-1.35)	1.13 (1.04-1.23)

CI: confidence interval

^a^All estimates are adjusted for age group, age group divided by exact age for each individual, gender, Charlson comorbidity index (low, medium, high), calendar period (1982-1986, 1987-1991, 1992-1996, 1997-2001, 2002-2003), a history of any autoimmune disease (yes/no), stage (localized, regional, distant, or unknown/missing), and the following index cancer treatments: no/symptomatic, chemotherapy, radiation therapy, hormone therapy, operation, and other/missing treatment

^b^Includes the phenotypic variant chronic lymphocytic leukemia

**Table 3:** Mortality rate ratios and 95% confidence intervals for death from cancer associated with various characterstics in persons diagnosed with cancer of the lung, colon, rectum, breast, prostate, or non-Hodgkin's lymphoma (NHL)^a ^in Denmark 1982-2003

*History of cutaneous squamous cell cacrcinoma*	1.13 (1.04-1.23)
*Male gender^b^*	1.27 (1.24-1.29)
*Age group (years)^c^*	
70-79	1.29 (1.26-1.31)
80-89	1.61 (1.57-1.65)
90-99	2.32 (2.15-2.50)
*Age difference^d^*	0.98 (0.98-0.98)
*Calendar period^e^*	
1987-1991	0. 97 (0.95-1.00)
1992-1996	0.87 (0.85-0.89)
1997-2001	0.65 (0.63-0.66)
2002-2003	0.49 (0.47-0.51)
*Comorbidity level^f^*	
Moderate	1.22 (1.19-1.24)
High	1.43 (1.37-1.49)
*Stage^g^*	
Regional	2.28 (2.22-2.33)
Distant	3.35 (3.26-3.44)
Unknown/missing	1.24 (1.21-1.28)
*Treatment of index cancer*	
No/symptomatic	0.93 (0.89-0.96)
Chemotherapy	1.01 (0.98-1.05)
Radiation	1.19 (1.16-1.23)
Operation	0.45 (0.44-0.47)
Hormone therapy	0.57 (0.55-0.59)
Missing/other	0.59 (0.56-0.62)
*Any autoimmune disease^h^*	1.07 (1.02-1.11)

^*a*^Includes the phenotypic variant chronic lymphocytic leukemia

^*b*^Reference is women

^*c*^Age reference is 50-69 years

^*d*^Continuous variable calculated as the midpoint of the age group divided by exact age for each individual

^*e*^Reference is the time period 1982-1986

^*f*^Reference is comorbidity level low

^*g*^Reference is localized stage

^*h*^Reference is no autoimmune disease

### Impact of prognostic factors

After including all other covariates, adding comorbidity to the model resulted in a 7% attenuation of the MRR for NHL, but had no effect in the remaining cancers. Adjusting for stage did not affect the results, while adjusting for cancer treatment had an impact in lung and breast cancer in the sense that it raised their MRRs by approximately 7%.

Increasing the enrollment period to 2008 resulted in no substantial change for prostate (MRR 1.03, 95% CI: 0.88-1.21) and colon cancer (MRR 1.10, 95% CI: 0.90-1.33), while an increase was observed for rectal cancer (MRR 1.43, 95% CI: 1.13-1.81) and NHL (MRR 1.23, (95% CI: 0.96-1.57).

## Discussion

We found that a history of cutaneous SCC was associated with a moderately increased mortality rate following a diagnosis of cancer of the lung, colon, rectum, breast, and NHL, but not in survivors of prostate cancer. The present study is the first to examine this association taking into account comorbidity and index cancer treatments. Adjustment for these factors had no substantial effect on our results, so it is unlikely that they have introduced major confounding in previous studies.

In agreement with the extant literature [4,10-13], we find an overall increased mortality rate in cancer patients with a history of cutaneous SCC. When examining index cancer types individually, there are, however, some inconsistencies with previous studies. Askling *et al*., Kahn *et al*., and Nugent *et al. *included prostate and breast cancer in their studies. All three studies found a 19 to 45% increased mortality rate among breast cancer patients with a history of cutaneous SCC. In comparison, we found a 9% increase. In contrast to Askling *et al. *and Kahn *et al*., we found no increased rate of death from prostate cancer. For the remaining index cancers, the finding of a poorer prognosis in patients with previous cutaneous SCC is consistent across studies [4,10-13]. In both colon and rectal cancer, only Kahn *et al. *found no increase in the mortality rate. This difference may be explained by the fact that they did not differentiate between NMSC subtypes, since Hjalgrim *et al. *and Nugent *et al. *showed that MRRs for these cancers were lower for BCC than SCC patients.

An association between a history of cutaneous SCC and a poor prognosis may be explained by an underlying immunodeficiency. Cumulative UVR exposure, the major risk factor of SCC of the skin [1], is especially interesting since UVR induces cellular immune incompetence [6]. This reduced immune function may be observed both locally and systemically and it is more pronounced in patients with previous skin cancer than in the general population [6,21]. We therefore hypothesize that cutaneous SCC is a marker of underlying immunosuppression that compromises the patient's normal immune surveillance against nascent tumor cells resulting in poor prognosis of subsequent cancer. NHL is interesting from this immunologic perspective, given its strong association with immune function. Hence, we would have expected a greater increase compared with the other cancers that are not as strongly related to immune function. However, the imprecision of our estimates, especially for NHL, limit us from concluding on any substantial differences between cancers. Furthermore, alternative explanations such as field cancerization due to an unidentified carcinogen may also explain our findings.

The low number of observations limited the analysis of associations for subtypes of autoimmune diseases, and since the degree of immunosuppression varies with severity and type of autoimmune disease "a history of any autoimmune disease" may be an imperfect reflection of immunosuppression. Furthermore, information on a history of autoimmune disease may be underreported, especially in the mild cases. These limitations prevent us from ruling out a differential effect in people with autoimmune disease.

Our study has several limitations. Even though the Danish Cancer Registry is close to complete for most malignancies [15], NMSC registration is probably underreported [5,22] due to the high incidence of NMSCs burdening the systems and the high cure rate that may cause clinicians to consider it trivial, especially in patients with comorbidity [23]. We do not, however, find it likely that mortality of a malignancy occurring later in time would affect misclassification of cutaneous SCC.

Misclassification of cause of death may be possible. The Danish Registry of Causes of Death has varying validity for different causes of death, although it is almost complete for cancer deaths [16,24,25]. The major problem with the registry is that the sequence of events may not be accurate [25]. To avoid this problem, we considered cause of death to be cancer if any cause on the death certificate was a malignancy. Moreover, we repeated all analyses for all-cause death by using the civil registration system, which is virtually complete [14]. This change in outcome slightly attenuated the results, but they were not substantially different from the cancer-specific MRRs.

Surveillance bias might have affected our results if preceding SCC of the skin is associated with increased medical surveillance leading to earlier diagnosis, and hence, better prognosis. This bias would have worked against the direction of the observed association, so it cannot explain the results. In addition, a Danish study of NMSC and risk of subsequent cancer found no evidence of more intensive surveillance for internal malignancies [5], which is in accordance with Danish guidelines on follow-up in patients with cutaneous SCC [26]. Moreover, we found no substantial difference between mortality rates stratified on time between SCC and index cancer diagnoses.

## Conclusions

The present study is the first to examine mortality in cancer patients with previous SCC of the skin, taking comorbidity and cancer treatments into account. Previous studies have found an increased risk of cancer in SCC patients [3-5] and our study extends this to also include an increased mortality of some types of cancer. Given the increasing incidence of cutaneous SCC [1], these results stress the importance of adherence to the existing recommendations of screening, diagnosis, and treatment of cancer in patients with a history of cutaneous SCC.

## Competing interests

The authors declare that they have no competing interests.

## Authors' contributions

SAJ, TLL, AØJ, and ABO made substantial contributions to the conception and design of the study. DKF performed the acquisition of data. SAJ conducted the analysis of the data. All authors contributed to the interpretation of data, drafting and revising the manuscript, and have given final approval of the version to be published.

## Pre-publication history

The pre-publication history for this paper can be accessed here:

http://www.biomedcentral.com/1471-2407/12/126/prepub

## Supplementary Material

Additional file 1 **International Classification of Diseases (ICD) codes used in the study**. A table presenting all ICD codes used in the study. Click here for file

Additional file 2 **Table with distribution of additional characteristics**. A table presenting the distribution of autoimmune diseases, stage, treatment, and the median, lower and upper quartile for the survival time in the cohort. Click here for file
