# Supplementary material for: Mortality in cancer patients with a history of cutaneous squamous cell carcinoma - a nationwide population-based cohort study
Source: BMC Cancer. 2012 Mar 29;12:126. doi: 10.1186/1471-2407-12-126 (PMC3337319; doi:10.1186/1471-2407-12-126)
Supplement: Additional file 1 — International Classification of Diseases (ICD) codes used in the study. A table presenting all ICD codes used in the study. [file 1471-2407-12-126-S1.PDF]

ADDITIONAL FILE 1: Table S1. International Classification of Diseases (ICD) codes used in the study

**Cutaneous Squamous Cell Carcinoma (SCC) and index cancers**	
Cutaneous SCC	ICD-10: C44 and morphological code: 80513, 80523, 80703, 80713, 80743, 80753, 80763, 80943, or 80953
Lung cancer	ICD-10: C34
Colon cancer	ICD-10: C18
Rectal cancer	ICD-10: C20
Breast cancer	ICD-10: C50
Prostate cancer	ICD-10: C61
Non-Hodgkin's lymphoma (including chronic lymphocytic leukemia)	ICD-10: C82-C85, C88, C90, C91, C96
**HIV and solid organ transplantation**	
HIV infection	ICD-8: 079.83, Y40.49, Y41.49; ICD-10: B20-B24, F02.4
Solid organ transplantation	ICD-8: 997.70, 997.79, Y95.09, Y95.89; ICD-10: T86.1-4, 94.0-4, Z94.8A; Danish classification of surgical procedures: 322.09, 322.29, 322.50, 356.09, 472.70, 472.79, 488.40, 488.49, 574.80, 574.90; NOMESCO Classification of Surgical Procedures: FQ, GDG, JLE, JJC, KAS
**Cause of death with ICD codes, grouped as defined by the National Board of Health into 14 categories**	
1. Tuberculosis, incl. sequelae	ICD-8: 010-019; ICD-10: A15-A19, B90
2. Infectious diseases excl. tuberculosis	ICD-8: 000-009, 020-136; ICD-10: A00-A09, A20-A99, B00-B89, B91-B99
3. Malignant tumors	ICD-8: 140-209; ICD-10: C00-D09
4. Dementia and stroke etc.	ICD-8: 290.09, 430-438, 440, 441, 442, 794; ICD-10: F03.9, I60-I72, R54
5. Heart disease	ICD-8: 390-398, 400-404, 410-414, 420-429; ICD-10: I00-I25, I27, I30-I52
6. Diseases of the respiratory tract	ICD-8: 460-474, 480-486, 490-493, 500-519; ICD-10: J00-J99
7. Diseases of the digestive organs	ICD-8: 520-577; ICD-10: K00-K93
8. Diseases of the urinary and reproductive organs	ICD-8: 580-629; ICD-10: N00-N99
9. Congenital Malformations and Chromosomal Anomalies	ICD-8: 740-759; ICD-10: Q00-Q99
10. Certain Conditions Originating in Perinatal Period	ICD-8: 760-779; ICD-10: P00-P96
11. Suicide	ICD-8: E950-959; ICD-10: X60-X84, Y87.0
12. Homicide	ICD-8: E960-E999; ICD-10: X85-Y09, Y87.1
13. Accidents etc.	ICD-8: E800-E807, E810-E823, E825-E949; ICD-10: V01-X59, Y10-Y86, Y87.2, Y88-Y89
14. All other causes of death	Remaining numbers
**ICD codes for autoimmune diseases**	
Hematological system	
Autoimmune hemolytic anemia	ICD-8: 283.90; ICD-10: D59.0, D59.1
Ideopatic thrombocytopenic purpura	ICD-8: 287.10; ICD-10: D69.3
Endocrine system	
Graves' disease	ICD-8: 242.00, 242.01, 242.08, 242.09; ICD-10: E05.0
Autoimmune thyroditis	ICD-8: 244.01, 245.03; ICD-10: E06.3
Addison´s disease	ICD-8: 255.10; ICD-10: E27.1
Diabetes type I	ICD-8: 249; ICD-10: E10
Central nervous/ neuromuscular system	
Multiple sclerosis	ICD-8: 340; ICD-10: G35
Myasthenia gravis	ICD-8: 733.09; ICD-10: G70.0
Gastrointestinal/hepatobiliary system	
Pernicious anemia	ICD-8: 281.00, 281.01, 281.08, 281.09; ICD-10: D51.0
Coelic disease	ICD-8: 269.00; ICD-10: K90.0

Crohn´s disease	ICD-8: 563.01, 563.02, 563.09; ICD-10: K50, M07.4
Ulcerative colitis	ICD-8: 563.19; ICD-10: K51, M07.5
Primary biliary cirrhosis	ICD-8: 571.90; ICD-10: K74.3
Skin	
Atopic dermatitis	ICD-8: 691.00; ICD-10: L20
Pemphigus/pemphigoid	ICD-8: 694.00-694.03, 694.05; ICD-10: L10.0, L10.1, L10.2, L10.4, L12.0
Dermatitis herpetiformis	ICD-8: 693.08, 693.09; ICD-10: L13.0
Psoriasis	ICD-8: 696.09, 696.10, 696.19; ICD-10: L40, M07.0-M07.3
Vitiligo	ICD-8: 709.01; ICD-10: L80
Connective tissue diseases	
Rheumatoid arthritis	ICD-8: 712.19, 712.29, 712.39, 712.59; ICD-10: M05, M06, G73.7D, I32.8A, I39.8E, I41.8A, I52.8A
Juvenile rheumatoid arthritis	ICD-8: 712.09; ICD-10: M08
Ankylosing spondylitis	ICD-8: 712.49; ICD-10: M45, H221B
Polymyositis/dermatomyositis	ICD-8: 716.09, 716.19; ICD-10: M33
Systemic- and subacute cutaneous lupus erythematosus	ICD-8: 734.19; ICD-10: M32, G05.8A, G73.7C, I32.8B, I39.8C, L93.1, L93.2, N08.5A, N16.4B
Systemic scleroderma	ICD-8: 734.00-734.09; ICD-10: M34.0-34.9
Mixed connective tissue disease	ICD-8: 734.91; ICD-10: M35.1
Sjögren´s syndrome	ICD-8: 734.90; ICD-10: M35.0, G73.7A, N16.4A
Sarcoidosis	ICD-8: 135.99; ICD-10: D86, G53.2, H22.1A, I41.8B, K77.8B, M63.3
Vasculitis syndromes including polymyalgia rheumatica	ICD-8: 287.09, 446.09-446.99; ICD-10: D69.0B, I77.6, L95, M30-M31, M35.3, M35.6, M79.3, N08.5B-N08.5E
Pulmonary system	
Idiopathic fibrosing alveolitis (pulmonary fibrosis)	ICD-8: 517.01; ICD-10: J841A, J841B, J841C
Ocular diseases	
Iridocyclitis	ICD-8: 364; ICD-10: H200, H201
Any autoimmune disease	If any of the codes listed above
