# Supplementary material for: Mortality in cancer patients with a history of cutaneous squamous cell carcinoma - a nationwide population-based cohort study
Source: BMC Cancer. 2012 Mar 29;12:126. doi: 10.1186/1471-2407-12-126 (PMC3337319; doi:10.1186/1471-2407-12-126)
Supplement: Additional file 2 — Table with distribution of additional characteristics. A table presenting the distribution of autoimmune diseases, stage, treatment, and the median, lower and upper quartile for the survival time in the cohort. [file 1471-2407-12-126-S2.PDF]

ADDITIONAL FILE 2: Table S2. Distribution of additional characteristics

Table S2. Selected characteristics of persons diagnosed with an index cancer (cancer of the lung, colon, rectum, breast, prostate, or non-Hodgkin's lymphoma (NHL)) in Denmark 1982-2003, by history of squamous cell carcinoma (SCC)

Characteristics	Lung cancer		Colon cancer		Rectal cancer	
	+ SCC (%)	– SCC (%)	+ SCC (%)	– SCC (%)	+ SCC (%)	– SCC (%)
Total	175	18,662	138	14,647	77	7,089
Any autoimmune disease	13 (7.4)	854 (4.6)	7 (5.1)	627 (4.3)	4 (5.2)	244 (3.4)
Survival time (years)						
Median	0.53	0.61	2.11	3.50	2.19	3.29
Lower quartile	0.20	0.23	0.70	0.96	0.94	1.13
Upper quartile	1.02	1.50	5.68	8.30	5.21	7.78
Time between diagnoses (years)[a]						
<1	23 (13)	–	23 (17)	–	10 (13)	–
1-4	55 (31)	–	46 (33)	–	26 (34)	–
>4	97 (55)	–	69 (50)	–	41 (53)	–
Stage						
Localized	62 (35)	5,663 (30)	62 (45)	7,447 (51)	42 (55)	3,639 (51)
Regional	47 (27)	5,993 (32)	49 (36)	4,627 (32)	17 (22)	2,021 (29)
Distant	38 (22)	4,321 (23)	13 (9.4)	1,844 (13)	7 (9.1)	777 (11)
Unknown/missing	28 (16)	2,685 (14)	14 (10)	729 (5.0)	11 (14)	652 (9.2)
Treatment of index cancer[b]						
No/symptomatic	94 (54)	7,513 (40)	8 (5.8)	575 (3.9)	11 (14)	459 (6.5)
Chemotherapy	16 (9.1)	4,012 (22)	3 (2.2)	1,010 (6.9)	1 (1.3)	258 (3.6)
Radiation	23 (13)	3,165 (17)	1 (0.72)	116 (0.79)	1 (1.3)	450 (6.4)
Operation	31 (18)	4,809 (26)	130 (94)	13,812 (94)	63 (82)	6,377 (90)
Hormone therapy	0 (0.00)	48 (0.26)	0 (0.00)	13 (0.090)	0 (0.00)	6 (0.08)
Missing/other	13 (7.4)	663 (3.6)	0 (0.00)	158 (1.1)	2 (2.6)	82 (1.2)

	Breast cancer		Prostate cancer		NHL[c]	
	+ SCC (%)	– SCC (%)	+ SCC (%)	– SCC (%)	+ SCC (%)	– SCC (%)
Total	113	10,418	186	21,364	56	6,963
Any autoimmune disease	9 (8.0)	437 (4.2)	9 (4.8)	853 (4.0)	5 (8.9)	322 (4.6)
Survival time (years)						
Median	4.93	6.57	2.97	3.07	2.41	3.50
Lower quartile	2.12	3.22	1.18	1.26	0.68	1.34
Upper quartile	7.97	10	5.09	5.94	5.27	6.63
Time between diagnoses (years)[a]						
<1	15 (13)	–	20 (11)	–	9 (16)	–
1-4	34 (30)	–	73 (39)	–	26 (46)	–
>4	64 (57)	–	93 (50)	–	21 (38)	–
Stage						
Localized	55 (49)	4,970 (48)	63 (34)	9,053 (42)	4 (7.1)	482 (6.9)
Regional	33 (29)	3,897 (37)	11 (5.9)	1,441 (6.7)	2 (3.4)	497 (7.1)
Distant	6 (5.3)	526 (5.1)	39 (21)	5,297 (25)	8 (14)	1,039 (15)
Unknown/missing	19 (17)	1,025 (9.8)	73 (39)	5,573 (26)	42 (75)	4,945 (71)
Treatment of index cancer[b]						
No/symptomatic	1 (0.88)	356 (3.42)	35 (19)	3,283 (15)	25 (45)	2,847 (41)
Chemotherapy	8 (7.1)	1,236 (12)	0 (0.00)	164 (0.77)	18 (32)	3,234 (46)
Radiation	12 (11)	2,336 (22)	7 (3.8)	939 (4.4)	6 (11)	473 (6.8)
Operation	97 (86)	9,492 (91)	99 (53)	13,210 (62)	1 (1.8)	151 (2.2)
Hormone therapy	25 (22)	2,763 (27)	28 (15)	3,564 (17)	0 (0.00)	19 (0.27)
Missing/other	2 (1.8)	79 (0.76)	25 (13)	2,078 (9.7)	7 (13)	625 (9.0)

[a] Time between SCC and index cancer diagnoses

[b] Numbers do not add up to 100% since some patients may receive a combination of therapies.

[c] Includes the phenotypic variant chronic lymphocytic leukemia
